# The relationship between intellectual curiosity and learning engagement in mathematics among adolescents: an analysis of chain multiple mediating effects

**DOI:** 10.3389/fpsyg.2025.1737254

**Published:** 2026-01-12

**Authors:** Yongzhao Wang, Lijun Zhou, Bingqing Xie, Lisha Wang, Qiantong Su, Hua Jin

**Affiliations:** 1 School of Mathematics and Statistics, Anyang Normal University, Anyang, China; 2 School of Management and Economics, North China University of Water Resources and Electric Power, Zhengzhou, China; 3 Department of Chinese Language and Literature, Gyeongsang National University, Jinju, Republic of Korea

**Keywords:** intellectual curiosity, learning engagement, multiple mediation model, school belonging, teacher support

## Abstract

**Introduction:**

Learning engagement (LE) is a core determinant of academic achievement and educational quality.

**Methods:**

Based on PISA 2022 data and focusing on a sample of adolescents in Korea (*N* = 6,308), the current study attempts to investigate how intellectual curiosity (IC) is associated with LE through multiple indirect pathways involving teacher support, encompassing universal, targeted, accompanying, and ongoing teacher support (UTS, TTS, ATS, OTS), and school belonging (SB).

**Results:**

The significant correlations were demonstrated of varying strengths among IC, UTS, TTS, ATS, OTS, SB, and LE. Further analysis indicated that IC not only influences LE directly, but also exerts indirect effects through UTS, TTS, ATS, OTS, and SB. Specifically, TS and SB exhibited both multiple parallel and multiple chain mediating effects in the relationship between IC and LE, thereby extending the self-determination theory. Additionally, subgroup analyses revealed that the mechanism exhibited significant and distinct divergence along the gender dimension. For instance, males reported a lower perception of TTS, whereas females' LE is more strongly shaped by the indirect effects of TS and SB.

**Discussion:**

These findings elucidate the complex mechanisms how IC affects LE, validate the four-dimensional structure of TS to extend SDT, offer a gender-sensitive intervention perspective for differentiated teaching, as well as provide practical insights for the holistic development of adolescents.

## 1 Introduction

Learning engagement (LE), as a crucial component of non-cognitive skills, holds a significant position in both educational theory and practice. Its predictive power encompasses not only cognitive performance but also non-cognitive variables such as mindset, self-efficacy, and perseverance, while simultaneously influencing students' academic achievement (Fredricks et al., 2004). LE emerges from the reciprocal transaction between individuals and environment, manifested as a transient and enjoyable state of focused learning that individuals experience when facing fewer demanding tasks (Bakker et al., 2017). Within the basic curricula, mathematics is widely regarded as one of the most cognitively taxing disciplines (Stodolsky et al., 1991). Mathematical LE is defined as the extent to which students actively engage in the acquisition of mathematical knowledge and employ cognitive strategies for deep thinking, constituting a robust predictor of achievement and a critical benchmark of instructional effectiveness (Reschly and Christenson, 2022). In spite of this, contemporary basic education mathematics classrooms predominantly feature monotonous teaching approaches. Students remain in a passive state of knowledge reception for extended periods, engaging minimally in the classroom. The phenomenon impedes sustained attention and effective cognitive activation, thereby constraining improvements in learning efficiency and quality (Freeman et al., 2014; Linnenbrink and Pintrich, 2003). Furthermore, despite the significant attention that LE has received, the relevant research into its formation mechanisms and enhancement pathways within the specific context of mathematics remains limited. The existing evidence is fragmented, and intervention studies require further development. Therefore, it is of significant theoretical and practical value to systematically explore the key factors that influence LE and the mechanisms that underpin it. Such exploration could help address the teaching dilemma of “low engagement-low achievement” and transform mathematics classrooms.

As a quintessential representative of East Asian education systems, Korea consistently ranks among the world's top performers in PISA mathematics literacy assessments, demonstrating outstanding academic achievement (OECD, 2023b). However, Korea's students endure intense academic pressure and score only 5.95 on the OECD's life satisfaction scale, placing Korea near the bottom among major nations and highlighting a pronounced imbalance between mental well-being and academic achievement (OECD, 2020). Concurrently, the substantial investment by Korean families in extracurricular tutoring reflects the systemic spillover of academic competition and the relentless accumulation of internalized pressure (Kim and Lee, 2010; Bray, 1999). In recent years, Korea has introduced education policies aimed at reducing school hours to alleviate student pressure. However, these measures have yielded limited results, with the scale of extracurricular education continuing to expand, which has created a unique educational tension characterized by “reduced in-school burden, increased out-of-school burden” (Kim, 2016; Golden and Figueroa, 2016). The educational ecosystem, characterized by high pressure and efficiency alongside a divergence between policy objectives and practical outcomes, provides a crucial empirical setting for investigating the mechanisms of learning that underlie academic achievement. Although the existing research has examined the impact of policy and environmental factors on the non-cognitive abilities of adolescents in East Asian countries (Zheng et al., 2025), there is a lack of empirical studies on the pathways to students' LE within the unique educational context of Korea. It is particularly noteworthy that aggregate national performance may obscure differences among distinct student groups. The existing research indicates that factors such as gender can significantly affect the mechanisms underlying LE by shaping different learning experiences, social expectations and access to resources (Yu et al., 2021). For example, male students often have greater self-efficacy in mathematics learning process, yet perceive less support (Lee and Seo, 2021; Kim and Alghamdi, 2023). Conversely, female students may rely more on external support systems, which may foster LE indirectly through emotional connections (Liu and Huang, 2025; Ren et al., 2025). Consequently, the current study attempts to focus on the mechanisms influencing LE among Korean adolescents, aiming to systematically elucidate the complex interplay among intrinsic motivation, teacher support and school belonging, while also conducting an in-depth analysis of gender-specific variations within the mechanism. Such exploration could provide a nuanced perspective and an empirical foundation for understanding learning processes within high-pressure educational contexts.

In the context of adolescent mathematics education, LE is recognized as a key indicator of academic achievement and deep understanding (Fredricks et al., 2004; Reschly and Christenson, 2022). Contemporary educational research indicates that LE was formed through a multidimensional, interactive process. The process requires not only intrinsic motivation for active participation, but also support and shaping from external environmental systems (Bronfenbrenner, 1975; Ryan and Deci, 2000). In order to systematically investigate the operational mechanisms of these factors, the present study takes an integrated approach, drawing upon self-determination theory (SDT) and social cognitive theory (SCT), and identifying three core variables that are representative of these theories. Intellectual curiosity (IC), originating from SDT, drives students' active exploration and deep engagement. Teacher support (TS) fulfils students' fundamental psychological needs through emotional care, cognitive guidance and sustained accompaniment, and acts as a crucial external environmental resource. School belonging (SB) reinforces students' identification with and emotional attachment to the school environment, acting as a mediator of socio-emotional connection. These three elements interact synergistically across intrinsic motivation, external support and socio-emotional dimensions to form an integrated support system that influences LE (Goodenow, 1993a; Roorda et al., 2011).

### 1.1 The foundational role of intellectual curiosity as an intrinsic motivator in learning engagement

Among the individual factors influencing students' development, IC is regarded as the primary motivator that drives proactive exploration and deep learning. It serves as a foundational element for learning behavior and cognitive engagement (Chu et al., 2021; Liu et al., 2024a). IC, defined as an intense desire and proactive pursuit of knowledge itself, is a psychological trait that combines cognitive drive and emotional engagement. It manifests when individuals derive enjoyment from exploring cognitive activities and seeking profound understanding of problems (Russell, 2013; Bluemke et al., 2024; Liu et al., 2024a). Theoretically, IC aligns closely with the framework of intrinsic motivation in SDT, wherein behavior is driven by the inherent interest and satisfaction derived from the activity itself, which was emerged as a key predictor of sustained student engagement in the learning process (Ryan and Deci, 2000). As a vital intrinsic driving force for students' learning process, IC has been extensively documented as a key force in stimulating learning motivation, fostering academic development, and enhancing educational quality (Powell et al., 2017; Orona and Pritchard, 2022; Ghosh, 2023; Von Stumm et al., 2011; Hidi and Renninger, 2006; Renninger and Hidi, 2015; Linnenbrink-Garcia et al., 2010). The research in workplace settings explored by Saeed AlShamsi et al. (2023) indicated that employees' cognitive curiosity significantly predicts their work engagement levels, subsequently stimulating innovative behavior. In an educational context, Vilhunen et al. (2022) found that cognitive curiosity effectively bridges the gap between theory and practice among science undergraduates by enhancing cognitive flexibility, thereby positively influencing academic performance. Specifically within the learning process, students with high levels of IC typically exhibit stronger learning initiative, including deeper cognitive engagement, more positive emotional involvement, and more persistent behavioral commitment. These characteristics collectively drive their comprehensive improvement in academic achievement, ability development, and mental health (Wu and Wu, 2020; Vracheva et al., 2020; Camacho-Morles et al., 2021). In summary, the prior and robust evidence suggests that IC may directly promote LE by stimulating students' intrinsic motivation to learn.

### 1.2 The teacher's bridge role between intrinsic motivation and learning engagement

From the perspective of the external environment, teachers, as organizers and implementers of teaching activities, exert a profound influence in the individual development of students. TS is typically described as the multi-layered assistance provided to students by educators through emotional care, cognitive guidance, and resource provision, aimed at fostering their learning and development. Additionally, TS comprises four complementary strands, including universal teacher support (UTS) extended to the entire cohort, targeted teacher support (TTS) tailored to individual needs, accompanying teacher support (ATS) that journeys alongside every learning moment, and ongoing teacher support (OTS) that sustains help without interruption (Trickett and Moos, 1973; Hamre and Pianta, 2001; Pianta and Hamre, 2009; Roorda et al., 2011). The theoretical underpinnings of the construct primarily stem from two classical perspectives. On the one hand, instructional interaction theory (IIT) characterizes it as observable behaviors within teacher-student classroom interactions, emphasizing its direct promotion of cognitive and social development (Hamre et al., 2013; Li et al., 2020; Zhu and Kaiser, 2022). On the other hand, SDT, originating from the fulfillment of basic psychological needs, interprets it as autonomy, competence, and relational support that can stimulate students' intrinsic motivation (Ryan and Deci, 2000; Guo et al., 2023). In recent years, large-scale international assessments such as PISA have shown a trend toward integrating both approaches. These approaches focus not only on the specific contexts of support, but also on students' subjective perceptions. They systematically evaluate four categories of support levels in order to capture the actual efficacy of teacher support more comprehensively (OECD, 2023a). The previous studies have inferred that TS is significantly associated with LE, indicating that students who perceive higher levels of tTS exhibit significantly greater behavioral, cognitive, and affective engagement in the learning process (Tao et al., 2022; Radulović et al., 2022; Affuso et al., 2023; Munk et al., 2025). Substantial empirical evidence further demonstrates that TS not only directly fosters multidimensional students' engagement, but also lays the foundational groundwork for sustained learning motivation by cultivating positive classroom environments and developing enduring teacher-student relationships (Roorda et al., 2011; Jang et al., 2016; Yang et al., 2018; He et al., 2025). Crucially, TS may mediate and amplify the relationship between motivation and behavior. It has been indicated by previous research that interactive teaching not only directly enhances learning outcomes but also strengthens students' cognitive understanding and mastery of teaching strategies by intensifying teacher-student interaction (Yang et al., 2022; Hettinger et al., 2023). To be specific, students with high IC are more inclined to proactively seek teacher guidance and resources, while the diverse forms of support provided by teachers subsequently translate into deeper and more sustained learning behaviors among students. The mechanism reveals that TS may not function as an isolated external variable but rather serves as a crucial bridge in the process of transforming students' intrinsic motivation for learning into observable learning behaviors.

### 1.3 The enhancing role of school belonging in the relationship between intrinsic motivation and actual behavior

Within the educational ecosystem, SB is a key indicator of the emotional bond formed between students and their institutional environment, playing an essential role in the process of maintaining learning motivation and encouraging sustained engagement (Goodenow, 1993b; Osterman, 2000). SB denotes a student's sense of identification with, emotional attachment to, and psychological security in the school as a social group, which reflects an individual's degree of social integration and emotional investment in the educational setting (Allen et al., 2018; Hagerty and Patusky, 1995). From the perspectives of social cognitive theory (SCT) and SDT, a strong sense of belonging satisfies students' relational needs and identity requirements, thereby providing deep emotional motivation and meaningful support for their learning behaviors (Ryan and Deci, 2000; Bandura, 2001). By fulfilling students' socio-emotional needs, a sense of belonging enhances their identification with and connection to the institution, thereby promoting deeper engagement in learning activities (Finn, 1989; Zukauskiene, 2017; Del Toro and Wang, 2022; Huang, 2022). Notably, SB directly fosters learning commitment by increasing classroom participation and persistence (Osterman, 2000; Goodenow, 1993a; Fredricks and Eccles, 2006), and it also plays a pivotal mediating role within the complex “motivation–environment–behavior” system. IC, the intrinsic force that drives students to explore knowledge, stimulates learning behaviors and reinforces students' identification with and sense of belonging to the school, which indirectly elevates their LE (Furrer and Skinner, 2003; Walton and Cohen, 2011), thereby further refining the dynamic influence pathway between SB and learning commitment (Dost and Mazzoli Smith, 2023; Osterman, 2000). Concurrently, TS can effectively enhance students' perceived sense of belonging through holistic, targeted and sustained approaches (Voelkl, 1995; Corcoran and Kelly, 2023; Wang and Eccles, 2012). Taken together, these findings suggest that SB may play a vital role in connecting IC and LE. SB may also work alongside TS to establish connections between IC and LE, helping to clarify the psychological processes through which students transform internal motivation into observable actions.

Although the prior research has examined the key networks that influence LE at three distinct levels such as individual motivation, interpersonal interactions and environmental adaptation, three significant limitations warrant further exploration. Firstly, despite the extensive study of LE, the previous research focusing on specific subjects (particularly mathematics) remains insufficient, making it difficult to determine the impact of subject differences on LE. Second, most existing studies concentrate on the influence of single factors on LE, lacking in-depth exploration of multiple mechanisms and thus failing to fully reveal the complex formation process of LE. Third, the relevant research on LE of Korean adolescents within the unique educational context of coexisting high academic pressure and educational reform aimed at reducing the academic burden is relatively scarce, yet the context provides a unique perspective for investigating the mechanisms of change in LE. Therefore, considering the importance of mathematics LE, the paucity of research on Korean adolescents, and the deficiency in detailed examinations of LE's mechanisms, the current study attempts to investigate the underlying mechanisms how IC, TS and SB collectively influence LE and also examine gender differences in the mechanisms. Such explorations could hopefully provide educational practitioners with scientific evidence and strategic recommendations, offer empirical support for developing differentiated educational policies and enhance the understanding for the relationship between IC and LE.

## 2 The present study

Although the previous studies have emphasized the positive impact of IC, TS and SB on LE, several research gaps still require urgent attention. Firstly, the prior research predominantly focuses on single factors or two-dimensional pathways, failing to examine the parallel and sequential mediating effects of four-dimensional TS and SB simultaneously. Secondly, there is virtually no empirical evidence concerning Korea's unique “high-pressure, reduced workload” educational ecosystem, which necessitates urgent supplementation. Thirdly, the relevant research focusing on subject-specific LE in mathematics is scarce, hindering the understanding of the mechanisms behind the low engagement-high achievement paradox in East Asian classrooms.

Building upon the prior research, the present study attempts to create an integrated model encompassing IC, four-dimensional TS, SB, and LE based PISA 2022 data for 6,308 Korean adolescents, further exploring the formation mechanisms of LE, aiming to address gaps in existing research. Specifically, the study's primary objectives are to: (1) assess the direct association of IC with adolescents' LE; (2) examine the multiple parallel indirect pathways of four-dimensional TS and SB in the relationship between IC and LE; (3) test the multiple sequential indirect pathways of four-dimensional TS and SB in the relationship between IC and LE; (4) investigate whether significant regional differences exist in the operational mechanisms of LE.

**Figure 1:**
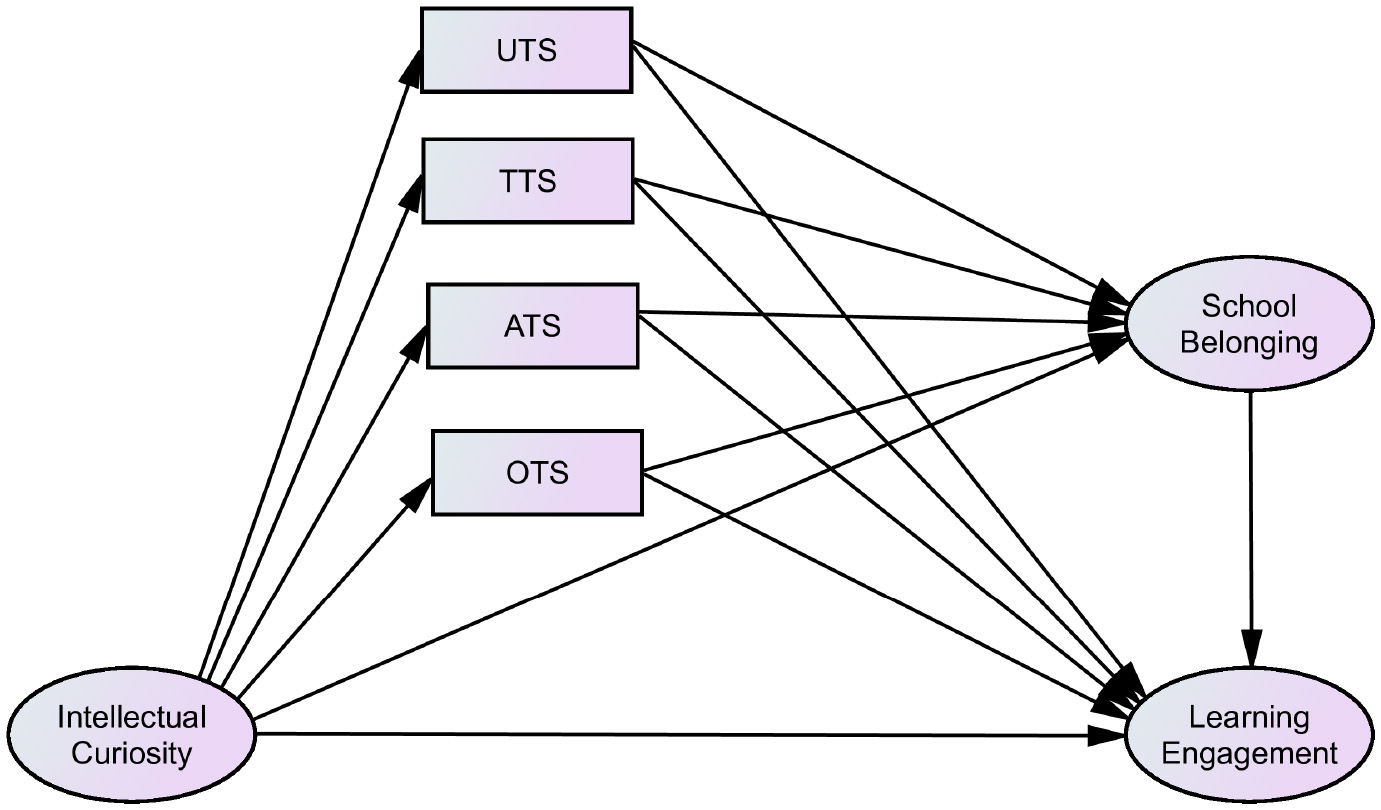
Hypothetical model.

Based on the above research content, the current study proposes the following hypotheses. The first research hypothesis (H1) posits that IC exerts a significant positive direct effect on LE. The second hypothesis (H2) indicates that four-dimensional TS and SB mediate the relationship between IC and LE through multiple parallel pathways. The third hypothesis (H3) posits that IC exerts a multiple chained mediating effect on LE through four-dimensional TS and SB. The fourth research hypothesis (H4) highlights that the influence mechanism of adolescents' LE exhibits significant gender differences. In summary, these research hypotheses aim to examine the influence mechanisms of students' IC, TS, and SB on LE, with the theoretical model illustrated in Figure 1.

## 3 Methods

### 3.1 Participants

The data analyzed in the present study are derived from PISA in 2022, administered by OECD. PISA is a global educational assessment project that primarily evaluates the literacy levels of 15-year-old students in the domains of mathematics, reading, and science. The focus of the PISA 2022 assessment was on mathematical literacy, with 81 countries or regions participating. Korea, as a typical representative of the East Asian education system, has consistently demonstrated outstanding performance in PISA assessments (OECD, 2023b; Golden and Figueroa, 2016). However, it also faces a unique educational landscape characterized by high academic pressure and ongoing educational reform aimed at reducing students' academic burden. The context provides a unique perspective for examining the mechanisms of change in students' LE, which is the primary reason for focusing on Korean adolescents in the current study. After listwise deletion of incomplete cases, the analytic file comprised 6,308 students nested in 397 schools. A total of 3,079 (48.80%) were females and the remaining 3,229 (51.20%) were males. The mean age was 15.77 (*SD*=0.264), and the average ESCS score for students was 0.239, which is on par with the OECD composite organization's average level.

### 3.2 Instruments

#### 3.2.1 Intellectual curiosity

The Intellectual Curiosity scale (ST301) from PISA 2022 was employed to assess students' IC. The scale comprises 10 items, including “I am curious about many different things”, and so on. Responses were captured on a 5-point Likert metric (1 = strongly disagree, 5 = strongly agree), and the two negatively worded items were reverse-coded before formal analysis. The OECD utilized weighted likelihood estimation (WLE) to fit the data based on students' responses regarding these 10 items. By setting the mean of students from OECD countries to 0 and the standard deviation to 1, the data were standardized to produce a composite index named “CURIOAGR”, which measures students' IC. In the present study, the scale demonstrated satisfactory reliability: Cronbach's α = 0.910 and McDonald's ω = 0.926. Confirmatory factor analysis (CFA) further indicated robust construct validity: χ^2^/df = 3567.610, CFI = 0.968, NFI = 0.968, IFI = 0.968, RMSEA = 0.092, TLI = 0.943, RMR = 0.029. Composite reliability (CR) and average variance extracted (AVE) were 0.914 and 0.527, respectively both within acceptable thresholds. Finally, the square root of AVE exceeded all inter-construct correlations, confirming adequate discriminant and convergent validity.

#### 3.2.2 Teacher support

The construction of TS was measured using four items from PISA 2022 that assess the teacher's support perceived by students, including UTS, TTS, ATS, and OTS (ST270). Rated on a 4-point frequency scale (“every lesson” to “never or almost never”), negatively keyed responses were reversed so that higher values denote stronger perceived support. For the purposes of the study, the scale achieved satisfactory reliability (Cronbach's α = 0.891; McDonald's ω = 0.925). CFA supported sound construct validity: χ^2^/df = 1807.120, CFI = 0.996, NFI = 0.996, IFI = 0.996, RMSEA = 0.066, TLI = 0.989, RMR = 0.008. CR (0.893) and AVE (0.676) both exceeded recommended thresholds, and the square root of AVE surpassed all inter-construct correlations, confirming satisfactory discriminant and convergent validity. Moreover, while PISA 2022 combined these four items into the teacher support scale, demonstrating sound validity and reliability at the aggregate level, the current study treated them as four distinct observed variables (UTS, TTS, ATS and OTS) within a structural equation model, exploring their distinct roles in the learning engagement mechanism. To confirm the independence of these four variables as parallel mediators, multicollinearity tests were conducted. The variance inflation factor (VIF) analysis revealed VIF values ranging from 1.08 to 2.83 for UTS, TTS, ATS and OTS, all of which were significantly below the commonly used critical threshold of 10. The result demonstrates that there are no severe multicollinearity issues and that they are suitable for inclusion as independent predictors within the model.

#### 3.2.3 School belonging

SB was assessed using the PISA 2022 School Belonging scale (ST034), consisting of six items, such as “I feel lonely at school”, and so on. All six items are measured on a four-point Likert continuum that ranges from 1 (strongly agree) to 4 (strongly disagree). Prior to analysis, items SB2, SB3, and SB5 were reverse-coded so that higher numeric values consistently represent a stronger SB. The OECD's responses to six items also yielded a composite index of SB, denoted as “BELONG”. For the current study, the scale demonstrated good internal consistency (Cronbach's α = 0.840; McDonald's ω = 0.885). CFA indicated robust construct validity: χ^2^/df = 1162.031, CFI = 0.994, NFI = 0.994, IFI = 0.994, RMSEA = 0.053, TLI = 0.985, RMR = 0.009. CR (0.839) and AVE (0.483) approached or met conventional thresholds, and the square root of AVE exceeded all inter-construct correlations, confirming good discriminant and convergent validity.

#### 3.2.4 Learning engagement

The PISA 2022 Mathematics Learning Engagement scale was utilized to gauge students' LE in the process of mathematics learning (ST293). The scale encompasses nine items, for example, “I actively participate in group discussions during mathematics class”, and so on. A five-point scale ranging from “never or almost never” to “always or almost always” was employed for assessment. Items LE4 and LE7 required reverse scoring prior to analysis, such that higher scores indicate greater student learning engagement. The “MATHPERS” composite indicator is likewise derived from responses to nine items by the OECD. The scale used in the present research exhibited strong internal consistency (Cronbach's α = 0.906; McDonald's ω = 0.924). CFA confirmed adequate construct validity: χ^2^/df = 3046.424, CFI = 0.977, NFI = 0.977, IFI = 0.977, RMSEA = 0.085, TLI = 0.964, RMR = 0.040. CR (0.911) and AVE (0.555) surpassed recommended thresholds, and the square root of AVE exceeded all inter-construct correlations, indicating satisfactory discriminant and convergent validity.

### 3.3 Data analysis

Given that the OECD employed a matrix sampling design to reduce students' response time when designing the questionnaire (OECD, 2023a), meaning that each student answered only a subset of questions. The method resulted in missing data rates of 45–56% for variables such as IC, SB, and LE in the current study. Little's MCAR test revealed that the data were not missing completely at random. To obtain unbiased parameter estimates, the study used the Expectation Maximisation (EM) algorithm to impute missing values (Khurma et al., 2025). Proposed by Dempster et al. (1977), the EM algorithm is a classical maximum likelihood estimation method for handling non-randomly missing data, whose efficacy has been extensively validated in structural equation modeling, item response theory and the internal data processing procedures of large-scale international assessments such as PISA (Mislevy and Wu, 1996; OECD, 2024). The method preserves data variability and associative structures more effectively than the simple imputation. All analytical results were subsequently weighted according to PISA's two-stage unequal probability sampling characteristics, with standard errors estimated using the repeated samples method to correct for sampling design bias.

All statistical analyses were performed in SPSS (version 26.0), structural equation modelling and mediation tests were conducted in AMOS (version 26.0) using maximum-likelihood estimation. The significance of each regression coefficient was assessed by a bias-corrected 95% confidence interval estimated from 5,000 bootstrap resamples, with an effect judged significant at the 0.05 level if the interval excluded zero.

## 4 Results

### 4.1 Common method bias

Harman's single-factor test was applied to all items measuring IC, the four dimensions of TS (including UTS, TTS, ATS, OTS), SB, and LE to assess common method bias (CMB). Seven factors exhibited eigenvalues greater than 1, with the first factor accounting for 29.021% of the total variance below the 40% threshold, indicating that CMB was within an acceptable range (Harman, 1976).

### 4.2 Descriptive statistics and correlation analysis of variables

To further investigate the potential relationships among IC, the four dimensions of TS, SB, and LE, descriptive statistics and Pearson correlation analyses were conducted for all variables, with the results presented in Table 1. It is evident that the mean IC score for Korean students is 0.091, with SD of 1.109, indicating substantial individual differences and exceeding the OECD average, thereby reflecting a certain degree of desire to explore new knowledge. The means of the four dimensions of TS range from 2.860 to 3.231, demonstrating that students perceive a moderate level of teacher support during their learning process. The mean SB score is 0.265, with SD of 1.002, also showing considerable individual variation, which suggests that students possess a relatively strong sense of identity with their school. The mean LE score stands at −0.127, below the overall average for OECD countries, indicating that students' commitment to mathematics learning is generally moderate. Furthermore, there are significantly positive correlations of varying degrees among IC, UTS, TTS, ATS, OTS, SB, and LE, collectively influencing students' learning enthusiasm and engagement levels.

**Table 1:** Descriptive statistics and correlation analysis of variables (*N* = 6308).

	**M**	**SD**	**1**	**2**	**3**	**4**	**5**	**6**	**7**
1. IC	0.091	1.109	1						
2. UTS	2.964	0.874	0.113**	1					
3. TTS	3.065	0.867	0.120**	0.677**	1				
4. ATS	3.231	0.821	0.116**	0.691**	0.737**	1			
5. OTS	2.860	0.937	0.080**	0.643**	0.626**	0.671**	1		
6. SB	0.265	1.002	0.253**	0.215**	0.197**	0.220**	0.194**	1	
7. LE	−0.127	1.123	0.288**	0.214**	0.197**	0.201**	0.201**	0.224**	1

^**^p < 0.01.

### 4.3 The construction and fitting of structural equation models

As a preliminary step, reliability and validity indicators were computed for each scale used in the present study. Cronbach's α, McDonald's ω, CR, and AVE all surpassed the widely accepted thresholds (McDonald, 1970; Fornell and Larcker, 1981; Kline, 2023), attesting to high internal consistency and validity. Univariate skewness and kurtosis for every item were then examined to assess normality, with detailed in Table 2. Absolute values of both indices remained below 2, meeting Collier (2020)'s criterion and confirming that the data approximate a normal distribution, thus satisfying the distributional assumptions for subsequent statistical analyses.

**Table 2:** Skewness and kurtosis of various variables.

**Variable**	**IC**	**UTS**	**TTS**	**ATS**	**OTS**	**SB**	**LE**
Skewness	0.985	−0.469	−0.586	−0.850	−0.321	−0.698	0.005
Kurtosis	1.537	−0.545	−0.458	0.058	−0.875	0.772	0.726

Subsequently, SEM was estimated via maximum likelihood with IC predicting LE through the four dimensions of TS (UTS, TTS, OTS, ATS) and SB as mediators. Model fit was evaluated across multiple iterations via modification indices and critical ratios. The final model achieved acceptable fit: χ^2^/df = 1893.353, CFI = 0.924, NFI = 0.924, IFI = 0.924, RMSEA = 0.067, TLI = 0.910, RMR = 0.063. The indices approximate conventional benchmarks, demonstrating satisfactory correspondence between the hypothesized model and the sample data (Hu and Bentler, 1999).

Consequently, the significance of the path coefficients was tested, with Table 3 summarizing the main results and detailing the extent to which each path coefficient was supported. All path coefficients reached statistical significance (*p* < 0.001), meaning that all hypotheses were accepted. Specifically, IC positively predicted SB (0.145), LE (0.255), and all four dimensions of perceived teacher support, including UTS (0.106), TTS (0.106), OTS (0.109), and ATS (0.079). Thus, students with higher IC reported greater SB, stronger LE, and higher levels of all teacher-support dimensions. In turn, UTS, TTS, OTS, and ATS, together with SB, significantly and positively predicted LE, indicating that both TS and SB foster students' engagement during the process of mathematics learning. Irrespective of whether the support is directed at the whole class, tailored to the individual, sustained across time, or provided concurrently with learning, it enhances students' SB and, consequently, their LE.

**Table 3:** Path analysis results.

**Path**	**Estimate**	**S.E**.	**β**	***p***
IC → UTS	0.125	0.002	0.106	***
IC → TTS	0.124	0.002	0.106	***
IC → ATS	0.120	0.002	0.109	***
IC → OTS	0.100	0.002	0.079	***
IC → SB	0.118	0.001	0.145	***
IC → LE	0.298	0.002	0.255	***
UTS → SB	0.059	0.002	0.085	***
TTS → SB	0.006	0.002	0.009	***
ATS → SB	0.085	0.002	0.115	***
OTS → SB	0.022	0.001	0.034	***
UTS → LE	0.077	0.002	0.078	***
TTS → LE	0.033	0.002	0.033	***
ATS → LE	0.065	0.003	0.061	***
OTS → LE	0.054	0.002	0.058	***
SB → LE	0.123	0.002	0.086	***

*** *p* < 0.001.

### 4.4 The analysis of multiple mediation effects

To examine the various mediating roles of UTS, TTS, OTS and ATS in conjunction with SB in the relationship between IC and LE, bias-corrected percentile bootstrapping was employed. The results, as presented in Table 4, demonstrated that IC not only shows a direct association with students' LE (β=0.255), but also exerts an indirect influence on LE through the mediating roles of UTS, TTS, ATS, OTS, and SB, with a total mediation effect of 0.038, accounting for 12.90% of the total effect. To be specific, the mediation effect values for the pathways IC → SB → LE, IC → UTS → LE, IC → OTS → LE, IC → ATS → LE, and IC → TTS → LE were 0.012, 0.008, 0.007, 0.005, 0.003 (effect sizes accounting for 4.30%, 2.80%, 2.30%, 1.60%, and 1.20% respectively). Moreover, the 95% confidence intervals for all five mediating paths did not span zero, with *p*-values reaching statistical significance, indicating the presence of five parallel mediating effects. The findings imply that UTS, TTS, OTS, ATS, and SB partially mediate the relationship between IC and LE, with SB demonstrating the strongest mediation effect followed by UTS, thereby confirming H1 and H2.

**Table 4:** The multiple mediating effects of teacher support and school belonging.

**Path**	**Effect**	**95%CI lower**	**95%CI upper**	***p*-value**	**Effect%**
Total effect	0.292	0.289	0.295	***	
Direct effect: IC → LE	0.255	0.251	0.258	***	87.10%
Total indirect effect	0.038	0.037	0.039	***	12.90%
IC → SB → LE	0.012	0.012	0.013	***	4.30%
IC → UTS → LE	0.008	0.008	0.009	***	2.80%
IC → TTS → LE	0.003	0.003	0.004	***	1.20%
IC → ATS → LE	0.005	0.004	0.005	***	1.60%
IC → OTS → LE	0.007	0.006	0.007	***	2.30%
IC → UTS → SB → LE	0.001	0.001	0.001	***	0.30%
IC → TTS → SB → LE	0.000	0.000	0.000	***	0.00%
IC → ATS → SB → LE	0.001	0.001	0.001	***	0.40%
IC → OTS → SB → LE	0.000	0.000	0.000	***	0.10%

***p < 0.001.

Additionally, regarding the chain-mediated effects, the indirect effects for paths IC → UTS → SB → LE and IC → ATS → SB → LE each equal 0.001, whereas paths IC → TTS → SB → LE and IC → OTS → SB → LE are around 0.000 at three decimal places. Collectively, these four paths accounted for 0.80% of the total effect. Although the effect sizes are extremely small, the *p*-values for all paths are less than 0.001, thus achieving statistical significance. These small yet significant chain-mediated pathways underscore that the influence of IC on LE is not exerted through a single direct route, but rather through a complex, multi-channel process, thereby offering a novel perspective on the intricate mechanisms underlying LE.

**Figure 2:**
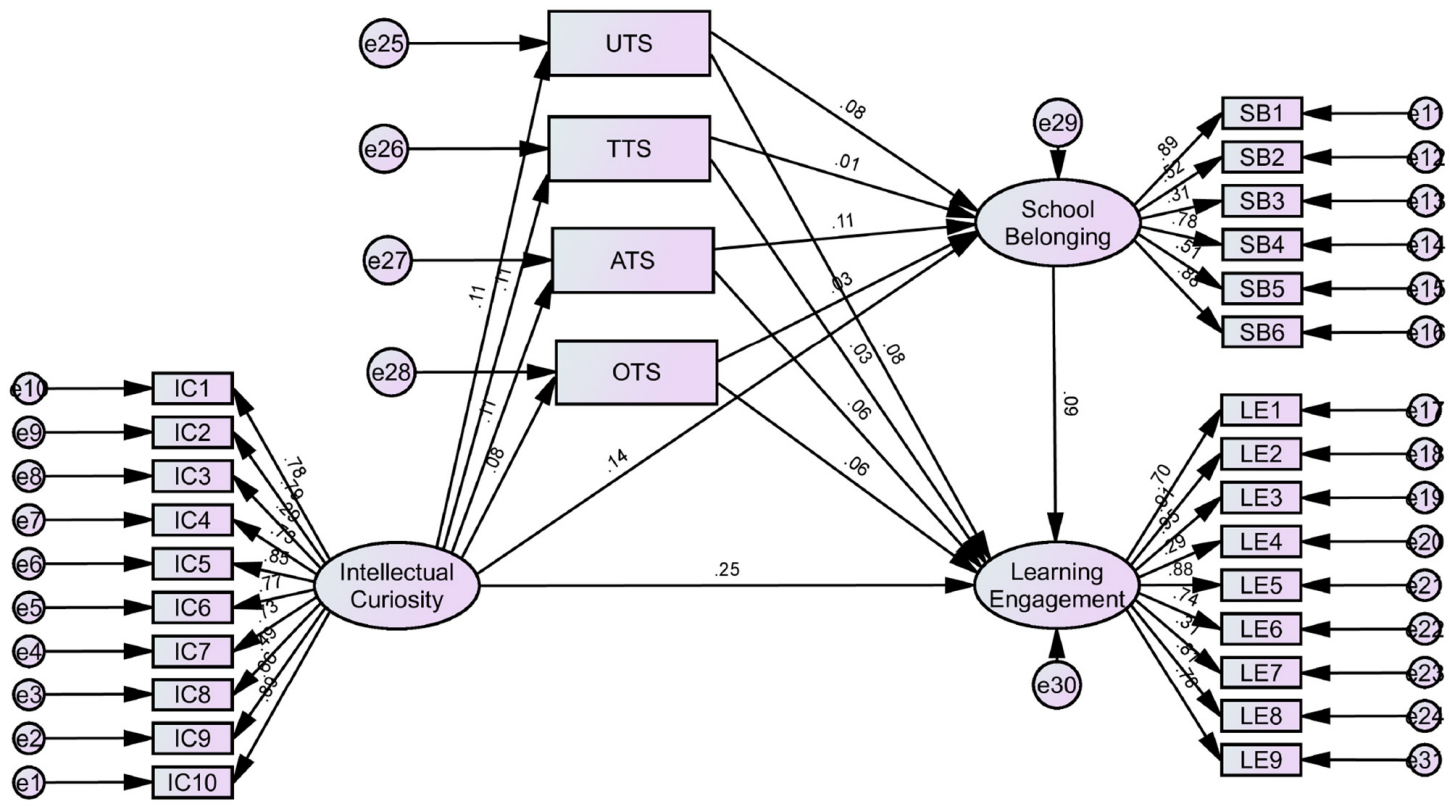
Path diagram for hypothetical model.

Overall, within the context of mathematics instruction, the influence of IC on LE can be categorized into three types of pathways. The first type is the direct effect pathway, where IC exerts a positive influence on LE. The second type includes indirect effect pathways, where IC positively impacts multiple mediating variables, such as UTS, TTS, OTS, ATS, and SB, which subsequently enhance LE. The third type comprises the chain mediating effect pathways, where IC initially positively affects variables like UTS, TTS, and OTS, ATS, which in turn positively influence SB, ultimately leading to increased LE. The specific structural model is depicted in Figure 2. The significance of the chain mediating pathways could enrich educators' understanding of the relationship between IC and LE and offer valuable guidance for educational practice.

### 4.5 The multi-group analysis of the mechanism underlying learning engagement

To investigate whether the complex mechanism by which Korean students' IC influences LE varies by gender, the current study employed a multi-group structural equation modelling analysis based on gender grouping to examine the stability of effect paths across groups. The results indicate that satisfactory overall fit indices were demonstrated by both male and female models. The adjusted fit indices for the male model are as follows: χ^2^/df = 1122.925, CFI = 0.918, NFI = 0.918, IFI = 0.918, RMSEA = 0.072, TLI = 0.904, RMR = 0.066. Those for the female model were: χ^2^/df = 1013.713, CFI = 0.910, NFI = 0.910, IFI = 0.910, RMSEA = 0.071, TLI = 0.895 and RMR = 0.060, suggesting that the model exhibits good fit across different gender groups (Hu and Bentler, 1999). Building upon the foundation, further tests for measurement and structural invariance were conducted. Using the unconstrained model (M1) as the baseline, constrained models were sequentially specified: M2 with equal measurement weights, and M3 with equal structural weights. The fit comparison results revealed significant differences between each constrained model and the baseline model, indicating that the theoretical model does not exhibit complete invariance across gender groups. Specifically, there are structural differences in the influence mechanisms of IC, UTS, TTS, ATS, OTS and SB on LE between male and female participants (see Table 5).

**Table 5:** The results of the multi-group analysis by gender.

**Model**	**χ^2^**	**df**	**χ^2^/*df***	**CFI**	**NFI**	**TLI**	**RMSEA**	***Δχ*^2^(Δdf)**	***p***
*M*_*male*_	389 654.843	347	1 122.925	0.918	0.918	0.904	0.072		
*M*_*female*_	351 758.403	347	1 013.713	0.910	0.910	0.895	0.071		
*M*_1_	741 413.244	694	1 068.319	0.914	0.914	0.900	0.051		
*M*_2_	750 410.722	720	1 042.237	0.913	0.913	0.902	0.050	8 997.478 (26)	0.000
*M*_3_	752 550.558	731	1 029.481	0.913	0.913	0.903	0.050	11 137.314 (37)	0.000

*M*_1_, unconstrained model; *M*_2_, measurement weights constrained; *M*_3_, structural weights constrained.

To examine whether there are significant differences in path coefficients between male and female groups, the present study conducted critical ratio (CR) analyses on path coefficients across multiple structural equation models (see Table 6). The CR is calculated as the difference between two parameter estimates divided by the standard error of that difference. Under large-sample conditions, the CR statistic approximates a standard normal distribution (Bollen, 1989). Consistent with common practice in behavioral science research, the current research adopts the criterion |CR|>1.96 to identify statistically significant gender differences in path coefficients at the α = 0.05 level (Abd-El-Fattah, 2010). It should be noted that within the context of a large sample (N=6308), even small between-group differences in standardized path coefficients (β) may attain statistical significance. Consequently, the present study will report statistically significant differences while also providing a comprehensive description incorporating the magnitude of coefficient differences. The results indicate that the CR values for the OTS → SB and OTS → LE pathways were −0.268 and 1.606, respectively, demonstrating no significant difference between the male and female groups and thus exhibiting stability (both values are less than 1.96). However, the CR values for all other pathways exceeded the critical range, indicating significant differences between the male and female samples. The influence of the IC → OTS, IC → ATS, IC → UTS, TTS → SB, TTS → LE, ATS → LE and SB → LE pathways are evidently more pronounced among female participants. Notably, the path coefficient for IC → OTS is 0.068 for males and 0.109 for females, indicating a more significant effect among females (CR < 0). Conversely, the effects of the paths IC → TTS, IC → SB, IC → LE, UTS → SB, ATS → SB, UTS → LE and OTS → LE are more pronounced among males. Notably, the path coefficient for IC → LE is 0.292 among males and 0.219 among females, indicating a more significant effect among males (CR > 0).

**Table 6:** The path differences in multi-group analysis based on gender.

**Path**	**Male**	**Female**	**CR**
IC → UTS	0.099***	0.134***	−11.771
IC → TTS	0.120***	0.114***	2.183
IC → ATS	0.112***	0.135***	−6.711
IC → OTS	0.068***	0.109***	−14.374
IC → SB	0.154***	0.138***	3.236
IC → LE	0.292***	0.219***	26.719
UTS → SB	0.104***	0.063***	8.233
TTS → SB	0.006	0.015***	−1.967
ATS → SB	0.071***	0.172***	20.386
OTS → SB	0.031***	0.033***	−0.268
UTS → LE	0.091***	0.071***	5.609
TTS → LE	0.004	0.056***	−10.698
ATS → LE	0.026***	0.090***	−12.762
OTS → LE	0.062***	0.061***	1.606
SB → LE	0.070***	0.102***	−7.733

***p < 0.001.

Moreover, as can be seen in Table 7, the mediation analysis reveals that IC exerts an indirect influence on LE among both male and female groups through the mediating effects of TS and SB, forming significant mediating pathways. However, it is notable that the proportion of direct effects in the male groups was 91.00%, which is significantly higher than the 80.60% observed in the female students. The result reflects the greater promotional role that IC itself plays in the cognitive mechanisms of male adolescents. Furthermore, TTS did not significantly influence SB or LE among males (*p*> 0.05). Consequently, the mediating effects along the paths IC → TTS → LE and IC → TTS → SB → LE were also insignificant, indicating that students' IC does not affect LE through the mediation of TTS. For female students, while the significance of each pathway aligns with the overall model, the proportion of total indirect effects relative to the overall effect is markedly higher than average. The difference suggests that the development of female students' LE relies more heavily on the indirect influence of TS and SB. In other words, TS and SB more effectively translate curiosity into engagement with the process of mathematics learning among female students. In summary, gender exerts a significant moderating effect on the complex mechanism through which IC influences LE, thereby supporting H4.

**Table 7:** The gender subgroup analysis of multiple mediating effects.

**Gender**	**Path**	**Effect**	***p***	**95%CI lower**	**95%CI upper**	**Effect %**
Male	Total effect	0.321	***	0.317	0.326	
	Direct effect: IC → LE	0.292	***	0.288	0.297	91.00%
	Total indirect effect	0.029	***	0.028	0.030	9.00%
	IC → SB → LE	0.011	***	0.010	0.012	3.40%
	IC → UTS → LE	0.009	***	0.008	0.009	2.80%
	IC → TTS → LE	0.001	0.214	0.000	0.001	
	IC → ATS → LE	0.003	***	0.002	0.004	0.90%
	IC → OTS → LE	0.004	***	0.004	0.005	1.30%
	IC → UTS → SB → LE	0.001	***	0.001	0.001	0.20%
	IC → TTS → SB → LE	0.000	0.154	0.000	0.000	
	IC → ATS → SB → LE	0.001	***	0.000	0.001	0.20%
	IC → OTS → SB → LE	0.000	***	0.000	0.000	0.00%
Female	Total effect	0.272	***	0.267	0.277	
	Direct effect: IC → LE	0.219	***	0.214	0.224	80.60%
	Total indirect effect	0.053	***	0.051	0.054	19.40%
	IC → SB → LE	0.014	***	0.013	0.015	5.20%
	IC → UTS → LE	0.010	***	0.009	0.010	3.50%
	IC → TTS → LE	0.006	***	0.006	0.007	2.30%
	IC → ATS → LE	0.012	***	0.011	0.013	4.50%
	IC → OTS → LE	0.007	***	0.006	0.007	2.40%
	IC → UTS → SB → LE	0.001	***	0.001	0.001	0.30%
	IC → TTS → SB → LE	0.000	***	0.000	0.000	0.10%
	IC → ATS → SB → LE	0.002	***	0.002	0.003	0.90%
	IC → OTS → SB → LE	0.000	***	0.000	0.000	0.00%

***p < 0.001.

## 5 Discussion

The current study attempts to examine the mechanisms through which Korean students' IC is linked to their LE, with particular attention to the multiple chain-mediating roles of four TS dimensions including UTS, TTS, ATS, OTS, and SB. The research has yielded four valuable findings.

The first study found a significant positive correlation between adolescents' IC and their engagement during the process of mathematical learning. Specifically, as IC increases, students exhibit heightened levels of cognitive, emotional, and behavioral investment in mathematical tasks. The phenomenon suggests that IC serves as a critical internal drive that encourages students to actively participate in the process of mathematics learning and to explore mathematical knowledge more deeply. The finding aligns with the principles of SDT, which asserts that learners with intrinsic interest and motivation might tend to participate meaningfully in academic activities (Deci and Ryan, 2013). SDT emphasizes that intrinsic motivation is the key factor in sustained, deep engagement. When students are motivated by curiosity and a desire to explore knowledge itself, their learning process exhibits greater autonomy and persistence. From a cognitive motivational perspective, students with strong IC tend to perceive mathematical learning as meaningful and challenging exploration rather than passive tasks. The intrinsic orientation prompts them to allocate more cognitive resources, demonstrate greater resilience when encountering difficulties and derive greater psychological satisfaction from understanding complex concepts. It was worthy that heightened IC correlates with the brain's reward system, particularly the release of dopamine (Kang et al., 2009; Jepma et al., 2012). As a neurotransmitter associated with pleasure, motivation and reward, dopamine is released during novel and challenging tasks, thereby enhancing motivation and self-efficacy (Murayama et al., 2010). Conversely, heightened self-efficacy fosters greater engagement and persistence in learning process, thereby elevating learning commitment (Sökmen, 2021). The promotional effect of IC on LE has received robust support through the data analysis of the current study, indicating that stimulating and cultivating students' IC should constitute a key focus for enhancing the quality of mathematics education.

The present study, through the structural equation modeling analysis, yielded a second significant finding, which is that the association between IC and LE is mediated through multiple pathways, with the mediating effect of SB being the most pronounced, followed by that of UTS. The finding empirically supports a core tenet of SDT, which posit that external environmental factors enhance intrinsic motivation by satisfying students' fundamental psychological needs, including autonomy, competence, and relatedness (Deci and Ryan, 2012). Specifically, the study reveals that, as a key manifestation of the need for relatedness, school belonging plays the most prominent role in stimulating students' learning motivation. Meanwhile, UTS indirectly fulfills students' autonomy and competence needs by fostering a fair and inclusive classroom atmosphere. When teachers provide timely feedback, acknowledge students' efforts and achievements, and offer personalized support, students are more likely to perceive their learning activities as supported, thereby enhancing their intrinsic motivation and LE. Together, the two pathways constitute the “psychological needs–motivation activation–behavioral engagement” transmission mechanism, which systematically explains how external environmental support transforms high-curiosity students into those who engage in sustained learning behaviors. The current research is valuable because it not only validates the applicability of SDT within the mathematics learning contexts, but also quantifies the specific contributions of different psychological need satisfaction mechanisms through mediated pathways. The conclusion provides precise theoretical foundations and practical directions for subsequent educational interventions.

The third research finding emerges from the multiple chain-mediation analysis, which elucidates the complex indirect pathways linking IC to LE. The results reveal that the impact of IC on LE is mediated in multiple ways. IC is first used to elevate the four dimensions of TS, including UTS, TTS, ATS and OTS. In turn, students' SB is enhanced by these, thereby translating curiosity into sustained LE. It can be seen that chain-mediation effects are empirically evident yet remain insufficiently documented in the extant literature, thereby constituting an innovation of the present study. While the effect size of the pathway is modest, it aligns with multi-step mediation theory (Hayes, 2017), which suggests that effects may diminish progressively as they are passed on. Nevertheless, given the continuity and cumulative nature of educational practice, these subtle chained effects may, in the long term, coalesce into a meaningful driving force for mathematics learning. The phenomenon occurs as a result of positive feedback loops that persist over time. Students receive TS due to their IC, which strengthens their sense of belonging and elevates their engagement levels (Rucker et al., 2011). The findings underscore that the influence mechanism of IC on LE is a multilevel, multifactorial process and offer a distinctive perspective for understanding how IC shapes adolescents' mathematics-related psychological states to impact LE. Future research could build on these findings by incorporating variables such as the quality of classroom interactions and individual student characteristics, in order to elucidate the indirect and mediating roles of TS in real educational settings more comprehensively (Hattie, 2008).

A more thorough investigation into gender disparities revealed that, in addition to shared mechanisms, the pathways to LE exhibited substantial and discrete variations between male and female students, constituting the fourth finding of the current study. Specifically, the mediating effects of the pathways IC → TTS → LE and IC → TTS → SB → LE were not statistically significant for male students, suggesting that TTS struggled to translate their IC into actual LE. Conversely, female students exhibited a complete chain of mediating pathways: IC → TS → SB → LE. The result reflects their greater reliance on external support systems to strengthen their sense of belonging on campus, thereby effectively converting their intrinsic motivation into learning behavior. Although other pathways (e.g., UTS → SB) exhibit statistically significant differences in coefficients, the disparities between groups in standardized β values remain relatively small. Therefore, their educational implications should be interpreted with caution within specific contextual frameworks. These gender-specific pathways may be rooted in the different societal expectations and educational practices for males and females in Korea. For example, the lack of a significant response from male students to TTS could be linked to the long-standing authoritarian parenting style in the Korean educational environment and specific expectations surrounding masculinity. In an education system that emphasizes academic competition and gender role differentiation, males are often implicitly expected to demonstrate innate advantages in core subjects such as mathematics. Such stereotypes may cause them to view TTS as a challenge to their abilities, reducing its effectiveness. As Patall et al. (2018) noted, when students perceive teacher support as excessive control, their intrinsic motivation may be suppressed. Concurrently, the significant mediating effect of female students in the pathway linking TS to SB may be closely tied to societal expectations in Korea for females to embody “relationship-oriented” and “cooperative” traits. Within the cultural emphasis on collective harmony, female students may be more inclined to interpret teacher support as an expression of emotional recognition and social inclusion. Their sense of belonging within the academic community were enhanced in turn and thereby promotes LE. It is worth emphasizing that the gender-differentiated pathways identified in the Korean sample offer an intriguing contrast and complement to research findings from the East Asian educational contexts, which have explored the impact of external resources such as educational subsidies on non-cognitive abilities (Zheng et al., 2025). Collectively, these findings suggest that within the macro-environment of East Asian collectivist cultures coexisting with high academic pressure, the mechanisms through which intrinsic motivation and external support systems influence adolescent development may manifest distinct characteristics across nations, shaped by variations in specific educational policies and societal expectations.

To be more specific, the mechanisms underlying gender differences can be interpreted in the following three ways. Firstly, gender role socialization may lead to distinct “help-seeking patterns” among male and female students. Under the cultural expectation of relationship orientation, female students are more likely to view teacher support as a reliable resource and use it to motivate themselves to learn. Conversely, male students, operating under gender norms emphasising “self-reliance and self-strength”, may perceive TTS as a threat to their autonomy. The result triggers psychological resistance, thereby diminishing the support's effectiveness. Secondly, gender differences in self-efficacy may further moderate the “support-engagement” relationship. Despite their generally lower mathematical self-efficacy, females indirectly maintain LE through pathways involving teacher support and a sense of belonging. Males, who have higher self-efficacy, have insufficient conversion mechanisms if TTS does not also enhance their sense of belonging. Thirdly, the way people think about gender in cultures where everyone is a member of a group may make these differences stronger, meaning that females may need more support to feel like they belong, while males may not need as much emotional connection. In summary, the gender-specific pathways identified in the current study offer initial insights into the mechanisms underlying LE in mathematics among Korean adolescents. The study also suggests that future research could empirically test these tentative explanations by measuring specific cultural and familial variables, such as parenting styles, gender role identification and collectivism tendencies. The approach would deepen the understanding of the relationship between gender and learning mechanisms, and provide crucial evidence for creating more inclusive and tailored teaching support systems in East Asian educational contexts.

Although the current study reveals the complex mechanisms through which IC, TS and SB influence LE, based on large-scale PISA data, the following limitations remain. Firstly, the reliance on cross-sectional PISA 2022 data restricts the capture of students' daily experiences and micro-interactions, and weakens causal inference due to the absence of time-series information. For example, the relationship between TS and LE could be affected by reverse causality or latent variable interference. Future longitudinal tracking designs or randomized controlled trials are required to clarify temporal precedence and the dynamic evolution of variables. Secondly, the current study offers speculative interpretations of certain findings such as gender-differentiated pathways by drawing upon the theoretical frameworks of “collectivist culture” and “gender role expectations.” However, constrained by database limitations, it was unable to employ specialized cultural orientation scales or gender role identity measures for direct measurement and validation of these constructs. Consequently, these interpretations require future empirical testing through the introduction of individual-level cultural psychological variables. Thirdly, while efficient, quantitative questionnaires are useful, they struggle to elucidate the multifaceted cultural connotations of TS in Korean contexts. Future research should adopt a mixed-methods approach, employing experiential sampling to collect real-time data on fluctuations in curiosity and engagement behaviors on a daily basis, while also utilizing focus interviews and classroom observations to deconstruct the deeper meanings of support, belonging and engagement within gendered and subject-specific contexts. The method will address the limitations of single-questionnaire surveys in terms of cultural sensitivity and the level of detail provided. Furthermore, while the single-item indicators used in this study to represent the four categories of teacher support provide clearer interpretation, they may not fully account for measurement error across dimensions. Future research could use multi-item scales to examine the reliability and validity of each support dimension more closely. Finally, the examination-oriented nature of mathematics may amplify the effect of IC. Subsequent research should conduct cross-disciplinary comparisons and multi-grade longitudinal cohort studies. Integrating classroom audio-visual coding with individual growth curve modeling will identify critical turning points in LE and subject-specific mechanisms, providing more targeted educational intervention strategies.

## 6 Conclusion

The current study focuses on a sample of Korean adolescents to explore the mechanisms through which IC is associated with LE, with a particular emphasis on the multiple chain mediating effects of four dimensions of UTS, TTS, ATS, OTS, and SB. The main results of the study are summarized as follows: (1) Adolescent' levels of IC and SB were above the OECD average, while TS perceived by them was at a moderate level, with ATS rated the highest among the four types. In contrast, adolescents' engagement in mathematics learning was found to be relatively modest. (2) All key variables showed significant positive correlations with LE, jointly contributing to adolescents' enthusiasm and involvement in mathematics learning. (3) IC impacted LE through both direct and indirect channels, with TS and SB serving as intervening mechanisms. Among them, SB demonstrated the strongest mediating effect, indicating its pivotal role in transforming their learning motivation into active engagement. (4) The association between IC and LE involves both multiple parallel mediation and multiple chain mediation effects, highlighting that the mechanism underlying LE is a complex, multilayered, and multifactorial process. Moreover, significant gender differences were observed in the mechanism influencing LE, underscoring the necessity of tailoring instructional interventions to individual and gender-specific profiles.

Drawing on the above findings, the following educational insights are proposed: (1) Incorporate game-based learning to stimulate students' intellectual curiosity. Educational psychology suggests that IC, an intrinsic driver of knowledge-seeking, lets students engage more actively in academic activities when their curiosity is strongly developed (Wade and Kidd, 2019; Gruber and Fandakova, 2021), which was also confirmed in the present study. By integrating game elements into mathematics lessons, teachers can create interactive and engaging environments through activities such as the One-Minute Number Guessing Game, which sparks students' desire for knowledge, thereby enhancing their engagement with learning. (2) Strengthen emotional education to enhance teacher support. The current research underscores the pivotal function of TS within the process of mathematical learning. Educators should adopt a student-centered educational philosophy and provide emotional support by establishing trusting and respectful relationships with their students. They may also employ diverse teaching methods, such as three-second differentiated instruction using red, yellow and green hand signals, thereby fostering an inclusive learning environment that encourages exploration. (3) Enhance school culture to strengthen students' sense of school belonging. Schools could display posters in corridors featuring mathematicians' early years and students' trial and error success stories. They could also broadcast weekly student-recorded mathematical anecdotes over the intercom and host end-of-term collaborative maths festivals involving teachers and students. By consistently embedding the belief that exploration is cool into the fabric of the school, from the walls to the activities, schools can significantly boost students' sense of belonging. (4) Fostering collaborative family-school development to jointly promote student growth. As advocated by research findings in educational psychology (Christenson, 2004; Minke and Anderson, 2005; Butler et al., 2022; Gormley et al., 2024), effective home-school cooperation is crucial for enhancing students' SB and learning quality, which can effectively foster the all-round well-being of both body and mind. Schools should institutionalize bidirectional communication channels including regular parent conferences, digital portfolios, and targeted home visits, and collaborate with families to co-construct learning goals. When students perceive convergent expectations and support from both home and school, their academic self-efficacy and intrinsic motivation are markedly strengthened. (5) Exploring AI-enabled pathways for precision educational support. The complex influence mechanisms revealed by the present study provide clear targets for the application of artificial intelligence technologies in educational settings. Building on the successful application of multimodal data fusion analysis techniques to student mental health contexts (Liu et al., 2024b), educational agents can be developed to dynamically identify students' non-cognitive states, such as their expression of classroom curiosity and emotional engagement. Based on the key pathway of teacher support–school belongingness, an AI-assisted decision-making system could be developed to offer personalized support strategy recommendations to teachers. For example, it could recommend more autonomous challenge tasks for male students who perceive weaker TTS or encourage female students requiring belongingness support to participate in collaborative learning opportunities. The approach would reinforce the motivation-support-belongingness continuum precisely.

Building on existing literature, the present study has made three interlocked contributions centered around the emerging concept of “mathematics learning engagement.” First, theoretical innovation was achieved by integrating, for the first time, PISA 2022's four-dimensional operationalization of teacher support including UTS, TTS, ATS, and OTS into a unified self-determination theory framework. Within the framework, a chained model specifying IC → multidimensional TS → SB → LE was constructed. By moving beyond the traditional reliance on one- or two-dimensional indicators of TS, the dynamic coupling between the satisfaction of relatedness and competence needs was explicated, thereby extending the explanatory capacity of SDT in complex educational ecologies. Second, methodological innovation was realized through the employment of structural equation modeling on a nationally representative sample of 6,308 Korean adolescents, enabling the simultaneous estimation of parallel and chained multiple-mediation pathways for all four TS dimensions within a single analytical framework. Subsequent gender-based subgroup analyses quantitatively revealed the nullification of the TTS path among male students and the amplification of chained mediation among female students, yielding a dimension-chain-gender triangulated modeling strategy that is replicable for cross-cultural and cross-group investigations of motivational mechanisms. Third, contextual and cultural innovation was evidenced through an investigation situated within Korea's high stakes testing environment that coexists with curricular emphases on holistic education. Collectivist gender norms were found to amplify the support–belonging–engagement chain: females, socialized to maintain group harmony, exhibited heightened sensitivity to TS, whereas males, enculturated into self-reliance, displayed diminished affective connection needs. By redressing the Western-centric bias prevalent in extant scholarship, culturally calibrated empirical evidence and policy implications for motivational interventions across East Asian educational systems were furnished.

## Data Availability

The original contributions presented in the study are included in the article/supplementary material, further inquiries can be directed to the corresponding author.
